# FMS-related tyrosine kinase 3 ligand (Flt3L)/CD135 axis in rheumatoid arthritis

**DOI:** 10.1186/ar4403

**Published:** 2013-12-06

**Authors:** Maria I Ramos, Samuel Garcia Perez, Saida Aarrass, Boy Helder, Pleun Broekstra, Daan M Gerlag, Kris A Reedquist, Paul Peter Tak, Maria C Lebre

**Affiliations:** 1 Department of Clinical Immunology and Rheumatology, Academic Medical Center/University of Amsterdam, K0-126 Meibergdreef 9, 1105 AZ, Amsterdam, the Netherlands; 2 Department of Experimental Immunology, Academic Medical Center/University of Amsterdam, K0-126 Meibergdreef 9, 1105 AZ, Amsterdam, the Netherlands; 3 University of Cambrigde, U.K. Trinity Lane, Cambridge CB2 1TN, UK; 4 ImmunoInflammation Therapy Area Unit, GlaxoSmithKline, Stevenage, U.K. Gunnels Wood Road, Stevenage Herts SG1 2NY, UK

## Abstract

**Introduction:**

The FMS-related tyrosine kinase 3 ligand (Flt3L)/CD135 axis plays a fundamental role in proliferation and differentiation of dendritic cells (DCs). As DCs play an important role in rheumatoid arthritis (RA) immunopathology we studied in detail the Flt3L/CD135 axis in RA patients.

**Methods:**

The levels of Flt3L in (paired) serum and synovial fluid (SF) were quantified by enzyme-link immunosorbent assay (ELISA). Expression of Flt3L and CD135 in paired peripheral blood mononuclear cells (PBMCs) and synovial fluid mononuclear cells (SFMCs) was quantified by fluorescence-activated cell sorting (FACS). The expression of Flt3L, CD135 and TNF-Converting Enzyme (TACE) in synovial tissues (STs) and *in vitro* polarized macrophages and monocyte-derived DCs (Mo-DCs) was assessed by quantitative PCR (qPCR). CD135 ST expression was evaluated by immunohistochemistry and TACE ST expression was assessed by immunofluorescence. Flt3L serum levels were assessed in RA patients treated with oral prednisolone or adalimumab.

**Results:**

Flt3L levels in RA serum, SF and ST were significantly elevated compared to gout patients and healthy individuals (HI). RA SF monocytes, natural killer cells and DCs expressed high levels of Flt3L and CD135 compared to HI. RA ST CD68^+^ and CD163^+^ macrophages, CD55^+^ fibroblast-like synoviocytes (FLS), CD31^+^ endothelial cells or infiltrating monocytes and CD19^+^ B cells co-expressed TACE. IFN-γ-differentiated macrophages expressed higher levels of Flt3L compared to other polarized macrophages. Importantly, Flt3L serum levels were reduced by effective therapy.

**Conclusions:**

The Flt3L/CD135 axis is active in RA patients and is responsive to both prednisolone and adalimumab treatment. Conceivably, this ligand receptor pair represents a novel therapeutic target.

## Introduction

Rheumatoid arthritis (RA) is a chronic, inflammatory, autoimmune disease characterized by persistent synovitis and hyperplasia of the joint synovium, development of pannus, and invasion of leukocytes into the joint followed by destruction of local articular components such as cartilage and bone [1,2]. In the RA synovium a variety of cell types can be found, specifically T cells, B cells, macrophages and dendritic cells (DCs) [3,4]. DCs derive from two sources: stem cells in the bone marrow, and precursor cells found in the circulation. In humans there are four major groups of DCs so far characterized: myeloid DCs (mDCs), plasmacytoid DCs (pDCs), migratory DCs such as Langerhans cells and dermal DCs, and monocyte-derived DCs (mo-DC) [5]. Although DCs represent a relatively small subset of immune cells, they are widely distributed throughout lymphoid and nonlymphoid tissues [6].

DCs have a crucial role in the initiation of primary immune responses. Individuals with autoimmune disease show a high number of aberrantly activated DCs either in circulation or in the autoimmune lesions, secreting large amounts of proinflammatory cytokines that mediate inflammation and differentiation of pathogenic T-helper type 1 and T-helper type 17 cells [7]. Rheumatoid synovial DCs have been described as having a more mature, differentiated phenotype, expressing high levels of HLA-DR, CD86 and nuclear RelB, and have been observed to associate with T cells in perivascular mononuclear cell aggregates surrounding the postcapillary venules, and in germinal center-like structures [8]. In addition, the RA synovium contains abundant immature mDCs and pDCs that express cytokines (interleukin (IL)-12, IL-15, IL-18, and IL-23), HLA class II molecules, and costimulatory molecules that are necessary for T-cell activation and antigen presentation [9]. In the synovial fluid (SF), DCs exhibit a semi-mature phenotype showing low levels of CD80 and CD83 expression [9]. An important sequel of continued antigenic stimulation via DCs is the formation of lymphoid structures at the site of inflammation. By coordinating the recruitment and/or activation of other immune cells, DCs can drive the generation of ectopic lymphoid tissues, as in the case of inflamed synovia in RA and systemic lupus erythematosus [10].

FMS-related tyrosine kinase 3 ligand (Flt3L) is crucial for steady-state pDC and mDC development. Mice lacking Flt3L have reduced numbers of DCs [11], as do mice that are deficient in signal transducer and activator of transcription 3 [12], which is an important molecule in the Flt3L signaling cascade. Conversely, administration of Flt3L to mice or humans leads to a dramatic increase in DC numbers both in lymphoid and nonlymphoid organs [13]. Flt3L is abundantly expressed in most human tissues, as a membrane-bound form and/or as a secreted form. Flt3L is initially synthesized as a membrane-bound protein, which must be cleaved to become a soluble growth factor. The extracellular domain alone has been shown to be sufficient for bioactivity [14]. Ectodomain shedding of Flt3L is metalloproteinase dependent and is mediated by tumor necrosis factor-converting enzyme (TACE) [15], a type 1 membrane protein belonging to a large family of transmembrane metalloproteases (a disintegrin and metalloprotease domain gene family) that was originally identified as the enzyme responsible for the cleavage of pro-tumor necrosis factor (TNF) alpha [16], but also has numerous additional substrates and functions, including a critical role in activating the ligands of the epidermal growth factor receptor, and in the modulation of immune reactions [17]. The receptor for Flt3L, CD135 is a transmembrane receptor tyrosine kinase expressed in bone marrow cells during the early stages of hematopoiesis [18], where it is involved in the control of maintenance, expansion, mobilization and differentiation of progenitor cells [19]. CD135 is required for DC homeostasis, and inhibition of CD135-mediated signals results in fewer DCs [20]. The effects of CD135 deficiency are most evident in the periphery, where this receptor is essential for the homeostatic expansion of DC progenitor populations in lymphoid organs [21].

Flt3L has been shown to accumulate in RA SF and induces arthritis when injected into healthy mouse knee joints. In addition, administration of Flt3L worsens experimental arthritis, while tyrosine kinase inhibitors that target CD135 alleviate experimental arthritis in mice models [22,23]. Given the relevance of Flt3L/CD135 in early hematopoiesis and DC generation, and possible involvement in RA, we characterized in detail the expression of both receptor and ligand in RA patients in comparison with healthy individuals (HI) and non-RA disease controls.

## Methods

### Patients and controls

Patients with RA diagnosed according to the 2010 criteria defined by the European League Against Rheumatism [24] were included in the study. Gout patients and HI were used as controls.

Peripheral blood mononuclear cells (PBMC) and synovial fluid mononuclear cells were isolated by gradient centrifugation with Lymphoprep (Axis-Shield PoPAS, Dieren, the Netherlands). Cells were frozen in fetal calf serum (Invitrogen, Breda, the Netherlands) containing 10% dimethyl sulfoxide (Sigma Aldrich, Zwijndrecht, the Netherlands) until further experimentation. SF samples were obtained by arthrocentesis of inflamed knee joints. Cell-free SF samples were stored at -80°C. Synovial tissue (ST) specimens were obtained during arthroscopy (2.7 mm arthroscope; Storz, Tuttlingen, Germany) under local anesthesia [25]. The samples were snap frozen *en bloc* in Tissue-Tek OCT (Miles Diagnostics, Elkhart, IN, USA). The frozen blocks were stored in liquid nitrogen. Cryostat sections (5 μm) were mounted on glass slides (Star Frost adhesive slides; Knittelgläser, Braunschweig, Germany). The glass slides were sealed and stored at -80°C until immunohistological analysis. Demographic and clinical data of the patients are presented in Table 1. All patients gave written informed consent before inclusion in the study, and the study was approved by the Local Ethics Committee of the Academic Medical Center, University of Amsterdam.

**Table 1:** Demographic and clinical characteristics of rheumatoid arthritis and gout synovial tissue immunohistochemistry

	**Rheumatoid arthritis (*n*** **= 15)**	**Gout (*n*** **= 12)**
Age (years)	56 ± 13	74 ± 17
Sex, female/male	13/3	2/10
Disease duration (years)	10.3 ± 14.4	2.2 ± 3.5
Swollen joint count	7 ± 5	2 ± 3
C-reactive protein (mg/l)	26.6 ± 21.2	18.2 ± 17.75
Disease Activity Score in 28 joints	5.0 ± 1.2	nd
Erythrocyte sedimentation rate (mm/hour)	40.2 ± 30.3	27.7 ± 19.2
IgM-RF (kU/l)	493 ± 1406	nd
Anti-CCP (kAU/ml)	2472 ± 3600	nd
Number taking NSAIDS (positive/negative)	9/15	5/12
Number taking corticosteroids (positive/negative)	0/15	0/12
Number taking DMARDs (positive/negative)	0/15	0/12
Number taking anti-tumor necrosis factor (positive/negative)	0/15	0/12

Data presented as mean ± standard deviation or number. anti-CCP, anti-cyclic citrullinated protein; DMARD, disease-modifying anti-rheumatic drug; IgM-RF, IgM rheumatoid factor; nd, not determined; NSAID, nonsteroidal anti-inflammatory drug.

### Enzyme-linked immunosorbent assay

Serum and SF levels of Flt3L were determined by enzyme-linked immunosorbent assay (R&D Systems, Abingdon, UK) following the manufacturer’s instructions. Demographic and clinical data of the patients used in each experiment are presented in Table 2. To determine the relationship between serum Flt3L levels and clinical response in RA patients, Flt3L serum levels were also measured in patients who started treatment with either a different regimen of glucocorticoids or adalimumab.

**Table 2:** Demographic and clinical characteristics of rheumatoid arthritis paired serum and synovial fluid (enzyme-linked immunosorbent assay)

	**Rheumatoid arthritis paired samples (*n*** **= 9)**
Age (years)	58 ± 14
Sex, female/male	5/2
Disease duration (years)	15 ± 6
Swollen joint count	4 ± 4
C-reactive protein (mg/l)	20 ± 25
Disease Activity Score in 28 joints	4.3 ± 2.5
Erythrocyte sedimentation rate (mm/hour)	31.1 ± 23.1
IgM-RF (kU/l)	117.25 ± 273.1
Anti-CCP (kAU/ml)	290.7 ± 447.9
Number taking NSAIDS (positive/negative)	1/9
Number taking corticosteroids (positive/negative)	6/9
Number taking DMARDs (positive/negative)	0/9
Number taking anti-tumor necrosis factor (positive/negative)	2/9

Data presented as mean ± standard deviation or number. anti-CCP, anti-cyclic citrullinated protein; DMARD, disease-modifying anti-rheumatic drug; IgM-RF, IgM rheumatoid factor; nd, not determined; NSAID, nonsteroidal anti-inflammatory drug.

### Patients treated with high-dose glucocorticoids

Nine patients from the active arm of a previously conducted, double-blind, randomized, placebo-controlled trial were treated with 60 mg oral prednisolone daily for 1 week followed by 40 mg prednisolone daily during the second week [26]. Flt3L serum levels were measured at baseline and after 2 weeks. In this study, response was defined as a decrease in Disease Activity Score in 28 joints ≥1.2 after 2 weeks of glucocorticoid (prednisolone) treatment.

### Patients treated with adalimumab

Baseline demographic and clinical features of patients from the larger open-label, prospective, single-center adalimumab clinical trial have been described previously [27]. Forty-eight patients were included for the present analysis. All patients received 40 mg adalimumab subcutaneously every other week, in combination with a stable methotrexate dose for at least 16 weeks. Use of oral glucocorticoids (prednisone ≤10 mg/day) was allowed. Clinical response at 16 weeks was determined according to the European League Against Rheumatism response criteria [24].

### Flow cytometry

The expression of specific markers was investigated in PBMC/synovial fluid mononuclear cells by fluorescence-activated cell sorting analysis after surface or intracellular staining with specific antibodies that were conjugated to different fluorescent dyes. For the extracellular staining, cells were washed in phosphate-buffered saline containing 1% bovine serum albumin and 0.02% sodium azide and were incubated with specific antibodies for 30 minutes at 4°C. Intracellular staining for Flt3L was performed after staining for surface markers was completed (T cell, B cell, natural killer (NK) cell and DC markers) using paraformaldehyde 4% as a fixation method followed by saponin permeabilization and Flt3L staining for 30 minutes at 4°C. For further details see Additional file 1. Flow cytometry was performed using a FACS CANTO (Becton Dickinson, Breda, the Netherlands) and analyzed with Flowjo analysis software (Tree Star, Ashland, OR, USA).

### Immunohistochemistry

Briefly, endogenous peroxidase activity was inhibited in the acetone-fixed sections by 0.1% sodium azide and 0.3% hydrogen peroxide in phosphate-buffered saline. Sections were stained using mouse monoclonal antibodies against CD68 (clone EBM-11; Dako, Heverlee, the Netherlands), CD163 (clone 5cFAT; BMA Biomedicals, Augst, Switzerland) or CD135 (clone BV10A4H2; eBiosciences, Vienna, Austria). Sections were sequentially incubated with a secondary horseradish peroxidase-labeled antibody, followed by horseradish peroxidase detection using the AEC kit (Brunschwig, Amsterdam, the Netherlands), and hematoxylin (Klinipath, Duiven, the Netherlands) as the counterstain. Parallel sections were incubated with isotype-matched and concentration-matched monoclonal antibodies as negative controls. After immunohistochemical staining, coded sections stained for CD135, CD68 or CD163 were analyzed in a random order by computer-assisted image analysis [28]. For all markers, 18 high-power fields were analyzed. Images were analyzed with the Qwin analysis system (Leica, Cambridge, UK).

### Immunofluorescence

Frozen ST sections were fixed in acetone and blocked with 10% human serum (Dako, Glostrup, Denmark), followed by incubation with mouse purified monoclonal antibody against TACE (R&D Systems ) for 1 hour. After washing with phosphate-buffered saline/bovine serum albumin 1%, sections were incubated with a secondary horseradish peroxidase-labeled antibody for 30 minutes. For detection of the first primary antibody, a biotin-conjugated tyramide signal amplification (PerkinElmer Life Sciences, Boston, MA, USA) was used followed by streptavidin Alexa 594 antibody (Invitrogen, Bleiswijk, the Netherlands). After blocking with 10% mouse serum (Dako), the sections were incubated with fluorescein isothiocyanate-labeled mouse monoclonal antibodies against CD68 (BioLegend, London, UK), CD163 (BioLegend), CD19 (eBiosciences), CD55 (Becton Dickinson), CD3 (eBiosciences), CD31 (eBiosciences), or purified rabbit polyclonal von Willebrand factor (Dako) followed by a secondary antibody labeled with Alexa 488. The slides were mounted with Vectashield containing diamidino-2-phenylindole (Vector Laboratories, Burlingame, CA, USA) and were analyzed on a fluorescent imaging microscope (Leica DMRA, Wetzlar, Germany) coupled to a charge-coupled device camera.

### Monocyte purification, macrophage and dendritic cell differentiation

PBMCs were isolated from volunteer donor blood buffy coats (Sanquin, Amsterdam, the Netherlands) by gradient centrifugation with Lymphoprep (Axis-Shield PoPAS), and monocytes were further isolated by Percoll gradient separation (GE Healthcare, Zeist, the Netherlands). Differentiation of monocytes into macrophages was performed in IMDM/10% fetal calf serum supplemented with 100 μg/ml gentamycin (Invitrogen), in the presence of granulocyte–macrophage colony-stimulating factor (5 ng/ml), macrophage colony-stimulating factor (25 ng/ml), interferon-gamma (IFNγ, 10 ng/ml) or IL-10 (10 ng/ml) (all from R&D Systems) for 7 days. mo-DCs were differentiated in IMDM/5% fetal calf serum supplemented with 100 μg/ml gentamycin (Invitrogen), in the presence of granulocyte–macrophage colony-stimulating factor (50 ng/ml) and IL-4 (100 ng/ml) for 6 days.

### Quantitative measurement of mRNA expression

Gene expression (mRNA) in synovial biopsies from RA and gout patients, polarized macrophages and mo-DC was assessed by quantitative polymerase chain reaction (qPCR) as described in detail in Table 3. Demographic and clinical data of the patients used in each experiment are presented in Table 3.

**Table 3:** Demographic and clinical characteristics of rheumatoid arthritis and gout synovial tissue (quantitative polymerase chain reaction)

	**Rheumatoid arthritis (*n*** **= 22)**	**Gout (*n*** **= 12)**
Age (years)	60 ± 8	62 ± 14
Sex, female/male	13/9	2/10
Disease duration (years)	13 ± 14	2.3 ± 1.7
Swollen joint count	7 ± 5	2 ± 3
C-reactive protein (mg/l)	20 ± 26	36 ± 31
Disease Activity Score in 28 joints	5.0 ± 1.2	nd
Erythrocyte sedimentation rate (mm/hour)	38 ± 31	35 ± 20
IgM-RF (kU/l)	464 ± 1366	nd
Anti-CCP (kAU/ml)	2197 ± 3475	nd
Number taking NSAIDS (positive/negative)	12/22	4/12
Number taking corticosteroids (positive/negative)	9/22	1/12
Number taking DMARDs (positive/negative)	12/22	0/12
Number taking anti-tumor necrosis factor (positive/negative)	6/22	0/12

Data presented as mean ± standard deviation or number. anti-CCP, anti-cyclic citrullinated protein; DMARD, disease-modifying anti-rheumatic drug; IgM-RF, IgM rheumatoid factor; nd, not determined; NSAID, nonsteroidal anti-inflammatory drug.

### Statistical evaluation

All analyses were performed using Prism software (GraphPad, La Jolla, CA, USA). Flt3L levels in paired serum and SF from RA patients and controls were compared using the Wilcoxon matched-pairs test and the Mann–Whitney *U* test, respectively. Results from qPCR and immunohistochemistry were analyzed using the Mann–Whitney *U* test. Results from fluorescence-activated cell sorting analysis and qPCR for polarized macrophages and mo-DCs were analyzed using the Kruskal–Wallis test. *P* <0.05 was considered statistically significant.

## Results

### Flt3L levels in RA serum, synovial fluid and synovial tissue are significantly elevated

**Figure 1:**
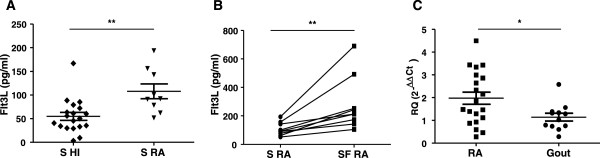
**FMS-related tyrosine kinase 3 ligand levels in rheumatoid arthritis serum, synovial fluid and synovial tissue are significantly elevated. (A)** FMS-related tyrosine kinase 3 ligand (Flt3L) serum levels (S) were increased in rheumatoid arthritis (RA; *n* = 9) compared with healthy individuals (HI; *n* = 19) (*P* = 0.0027). **(B)** Flt3L serum levels were elevated in RA synovial fluid (SF) compared with paired serum (*n* = 9) (*P* = 0.0019 **(C)**. Gene expression analysis by quantitative polymerase chain reaction showed increased Flt3L expression in RA synovial tissue (ST; *n* = 22) compared with gout ST (*n* = 12) (*P* = 0.0450). Each data point represents a single subject. Results presented as mean ± standard error of the mean mRNA expression of Flt3L relative to GAPDH. *P <0.05, **P <0.01.

We found significantly higher levels of Flt3L in serum of RA patients compared with HI (Figure 1A, *P* = 0.0027). In addition, there was a statistically significant increase (*P* = 0.0019) in the Flt3L levels in SF of RA patients compared with paired serum (Figure 1B). Consistent with increased activation of the Flt3L/CD135 axis in the synovial compartment of RA patients, we found increased mRNA levels of Flt3L in RA ST compared with gout ST (Figure 1C, *P* = 0.045). These differences could not be attributed to inflammation status since RA and gout patients were matched for IL-6 and IL-8 inflammatory parameters (Figure S1C,D in Additional file 2).

### Rheumatoid arthritis synovial fluid monocytes, natural killer cells and dendritic cells express high levels of Flt3L

**Figure 2:**
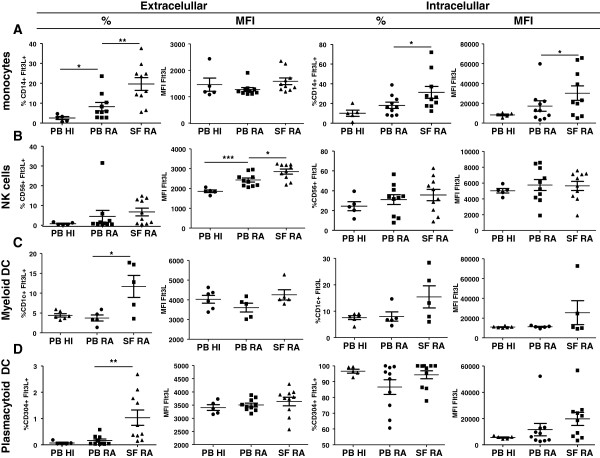
**Characterization of FMS-related tyrosine kinase 3 ligand expression in rheumatoid arthritis paired peripheral blood mononuclear cells (PBMC) and synovial fluid mononuclear cells and in healthy individual PBMC.** Intracellular and extracellular expression of FMS-related tyrosine kinase 3 ligand (Flt3L) by all cell types shown in terms of percentage or mean fluorescence intensity (MFI) (cellular marker^+^Flt3L^+^). **(A)** Percentage of extracellular Flt3L by CD14^+^ monocytes was significantly higher in rheumatoid arthritis (RA) peripheral blood (PB) compared with healthy individuals (HI; *n* = 10 and *n* = 5 respectively, *P* = 0.0400) and in RA synovial fluid (SF) compared with paired PB (*n* = 10, *P* = 0.0083). The percentage of intracellular Flt3L by CD14^+^ monocytes was increased in RA SF compared with paired PB. SF monocytes express higher Flt3L (MFI) compared with RA PB (*P* = 0.0318 and *P* = 0.0291 respectively). **(B)** Expression of extracellular Flt3L (MFI) by CD56^+^ natural killer (NK) cells in RA SF was significantly higher (*P* = 0.0191) compared with paired PB. CD56^+^ NK cells in RA PB expressed significant higher levels of extracellular Flt3L compared with HI peripheral blood mononuclear cells (PBMC; *P* = 0.0007). **(C)** CD1c^+^ myeloid dendritic cells (mDC) in RA SF expressed higher extracellular Flt3L compared with paired RA PB (*P* = 0.0317). **(D)** CD304^+^ plasmacytoid DC in RA SF expressed significant higher (*P* = 0.0015) levels of extracellular Flt3L compared with paired PB. Bars represent mean (± standard error of the mean) of five to 10 RA patients and HI. *P <0.05, **P <0.01.

To investigate the sources of Flt3L in peripheral blood (PB) and SF we characterized the relative expression of Flt3L on CD14^+^ monocytes, CD19^+^ B cells, CD56^+^ NK cells, CD4^+^ and CD8^+^ T cells and both CD1c^+^ mDCs and CD304^+^ pDCs present in RA paired synovial fluid mononuclear cells and PBMC and in HI PBMC by flow cytometry. During normal hematopoiesis, Flt3L is expressed constitutively but most of it is retained intracellularly within the Golgi apparatus [29]. We characterized in detail both intracellular and extracellular expression of Flt3L. We observed that the percentage of extracellular Flt3L on CD14^+^ monocytes was significantly higher (Figure 2A, *P* = 0.0083) in RA SF compared with PB. In addition, the percentage of PB RA CD14^+^ monocytes that expressed extracellular Flt3L was significantly higher (Figure 2A, *P* = 0.04) compared with HI PB.

The mean fluorescence intensity (MFI) is a measurement of expression per cell basis. In this respect, no differences were observed in extracellular Flt3L (expressed as MFI) on CD14^+^ monocytes from RA PB compared with paired SF (Figure 2A). We observed an increase in the percentage of intracellular Flt3L by CD14^+^ monocytes in RA SF compared with RA PB. Moreover, SF monocytes had a higher capacity to express Flt3L (MFI) compared with RA PB (Figure 2A, *P* = 0.0318 and *P* = 0.0291 respectively). The percentage of CD56^+^ NK cells that expressed Flt3L extracellularly or intracellularly was similar in HI and RA patients (Figure 1B). The expression of extracellular Flt3L (per cell basis, MFI) by CD56^+^ NK cells in RA SF was significantly higher (Figure 1B, *P* = 0.0191) compared with paired PB. In addition, in CD56^+^ NK cells RA PB expressed significantly higher levels of extracellular Flt3L compared with HI PBMC (Figure 1B, *P* = 0.0007). CD1c^+^ mDCs in RA SF expressed higher extracellular Flt3L compared with paired RA PB (Figure 2C, *P* = 0.0317). No differences were observed in the percentage of CD1c^+^ mDC-expressing Flt3L intracellularly or extracellularly in RA patients compared with HI, and no differences were observed in the levels expressed on a per cell basis. In RA SF, CD304^+^ pDCs expressed significantly higher (Figure 2D, *P* = 0.0015) levels of extracellular Flt3L compared with paired PB. No differences were observed on a per cell basis for intracellular Flt3L expression.

T cells contain intracellularly stored Flt3L but express low levels of extracellular Flt3L at the cell surface, as shown by flow cytometry analysis (Figure S1A,B in Additional file 2; see also [30]). Here we did not observe statistically significant differences in the percentage or level expressed on a per cell basis of Flt3L by both CD4^+^ and CD8^+^ T cells in PB and (paired) SF (Figure S3A,B in Additional file 3). The expression of Flt3L by CD19^+^ B cells was not significantly different between HI and RA PB or between paired RA PB and SF (Figure S3C in Additional file 3).

### CD135 is expressed in RA, gout and healthy individual synovial tissue

**Figure 3:**
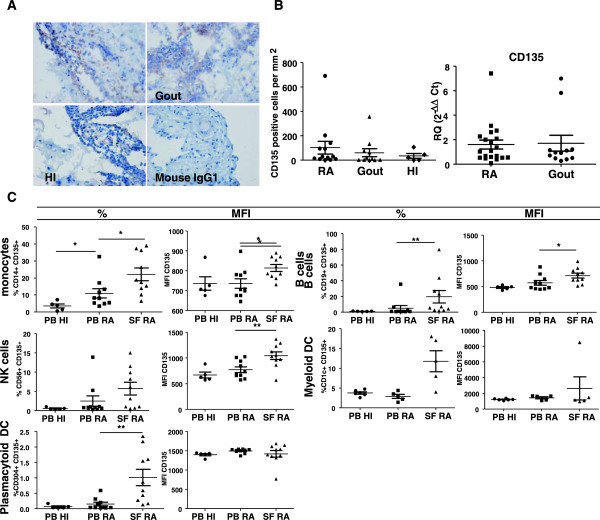
**CD135 expression in healthy individuals and rheumatoid arthritis and gout patients. (A), (B)** No differences were observed in CD135 expression in rheumatoid arthritis (RA) patient (*n* = 12), gout patient (*n* = 11) and healthy individual (HI; *n* = 7) synovial tissues (STs) by immunohistochemical analysis. **(B)** Right: CD135 gene expression in STs of RA (*n* = 22) and gout (*n* = 12) patients by quantitative polymerase chain reaction. Results presented as mean ± standard error of the mean (SEM) mRNA expression of CD135 relative to GAPDH. No differences were observed. **(C)** CD135 expression on CD14^+^ monocytes, CD56^+^ natural killer (NK) cells, CD304^+^ plasmacytoid dendritic cells (DC), CD19^+^ B cells and CD1c^+^ myeloid DC by flow cytometry. The percentage and mean fluorescence intensity (MFI) of CD135^+^CD14^+^ monocytes was higher in RA peripheral blood (PB) compared with HI PB (*P* = 0.0115 and *P* = 0.0260 respectively). In RA PB, CD135^+^CD56^+^ NK cell MFI was higher compared with HI PB (*P* = 0.0115). There were no differences for percentage or MFI of CD135^+^CD1c^+^ myeloid DC in RA compared with HI, or RA PB compared with paired synovial fluid (SF). The CD135^+^CD304^+^ plasmacytoid DC percentage was higher in RA SF compared with paired PB (*P* = 0.0029). Both the percentage and MFI of CD135^+^CD19^+^ B cells were higher in RA SF compared with paired PB (*P* = 0.0068 and *P* = 0.0491 respectively). Graphs represent mean ± SEM of five to 10 RA patients and HI. *P <0.05, **P <0.01.

Representative photomicrographs of CD135 expression (reddish-brown staining) in RA, gout and HI STs are shown in Figure 3A. The expression of Flt3L receptor CD135 in STs was similar between RA, gout and HI as quantified by digital image analysis (Figure 3B). In addition, we also measured mRNA expression of CD135 in RA and gout ST and there was no difference in expression between the two diseases, which confirmed the protein data obtained by immunohistochemistry (Figure 3B, right panel). We also performed immunohistochemistry to detect CD68^+^ and CD163^+^ macrophages to assess the level of synovial inflammation and measured IL-6 and IL-8 levels by qPCR. We observed that RA and gout patients were matched for these inflammatory parameters (Figure S1C,D in Additional file 2).

### Rheumatoid arthritis synovial fluid monocytes, NK cells and plasmacytoid DC express high levels of CD135

The percentage of CD14^+^ monocytes expressing CD135 was significantly (Figure 3C, *P* = 0.0115) higher in RA PB compared with HI PB. In addition, the expression of CD135 by CD14^+^ monocytes was significantly higher in RA SF compared with paired PB both expressed as a percentage of positive cells (Figure 3C, *P* = 0.0115) or as MFI (*P* = 0.0260). In RA PB, CD56^+^ NK cells showed significantly (Figure 3C, *P* = 0.0115) elevated expression (based only on MFI) of CD135 compared with HI PB. There were no differences in the percentage of positive cells or MFI of CD1c^+^ mDC expressing CD135 in RA compared with HI, nor in RA PB compared with paired SF. The percentage of CD304^+^ pDC expressing CD135 was significantly higher in RA SF compared with paired PB (Figure 3C, *P* = 0.0029) while no differences were observed in terms of MFI. We observed that both the percentage and expression per cell basis (MFI) of CD135 by CD4^+^ or CD8^+^ T cells did not differ between RA and HI PB nor between RA SF and paired PB (data not shown). In contrast with the results obtained for Flt3L, the expression of CD135 by CD19^+^ B cells was significantly higher in RA SF compared with paired PB, both in terms of percentage of positive cells (Figure 3C, *P* = 0.0068) and MFI (Figure 3C, *P* = 0.0491).

### CD68^+^ and CD163^+^ macrophages, CD55^+^ fibroblast-like synoviocytes, CD31^+^ endothelial cells and CD19^+^ B cells express TACE in RA synovial tissue

**Figure 4:**
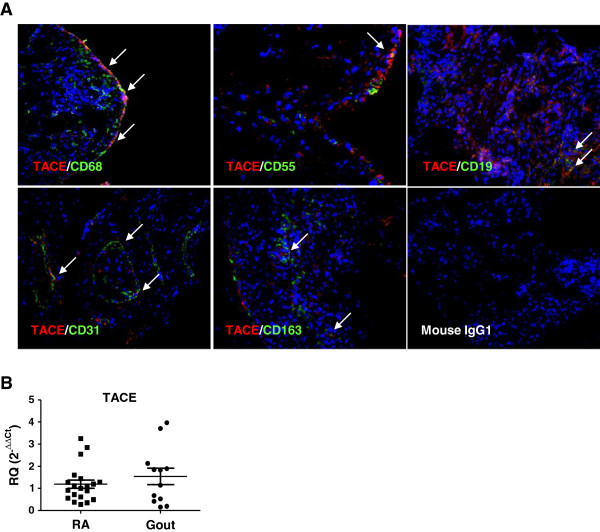
**Double immunofluorescence staining of TACE**^**+ **^**cells in rheumatoid arthritis synovial tissue. (A)** TACE^+^ single cells (red) and other cellular markers (green) can be seen. Tumor necrosis factor-converting enzyme (TACE) was colocalized (yellow, arrows) with CD68^+^ and CD163^+^ macrophages, CD55^+^ fibroblast-like synoviocytes, CD19^+^ B cells and CD31^+^ endothelial cells. Figures are representative of five rheumatoid arthritis (RA) patients. **(B)** Gene expression analysis of TACE mRNA by quantitative polymerase chain reaction (qPCR) in RA (*n* = 22) and gout (*n* = 12) synovial tissue analyzed by qPCR. Results presented as mean ± standard error of the mean mRNA expression of TACE relative to GAPDH. No significant differences were observed.

Flt3L can be proteolytically cleaved from the cell membrane by TACE [15]. In order to investigate the cellular source(s) of TACE in RA ST, immunofluorescence analysis was performed. TACE was expressed by several cell types in the RA synovium (Figure 4A; and Figure S2A in Additional file 4). These included CD68^+^ intimal macrophages, CD55^+^ fibroblast-like synoviocytes, CD31^+^ endothelial cells or infiltrating monocytes and CD19^+^ B cells (Figure 4A). CD3^+^ T cells and vWF^+^ blood vessels did not express TACE (Figure S2B in Additional file 4). In addition, TACE expression levels (mRNA) in RA and gout ST were similar (Figure 4B).

### IFNγ-differentiated macrophages express high levels of Flt3L

**Figure 5:**
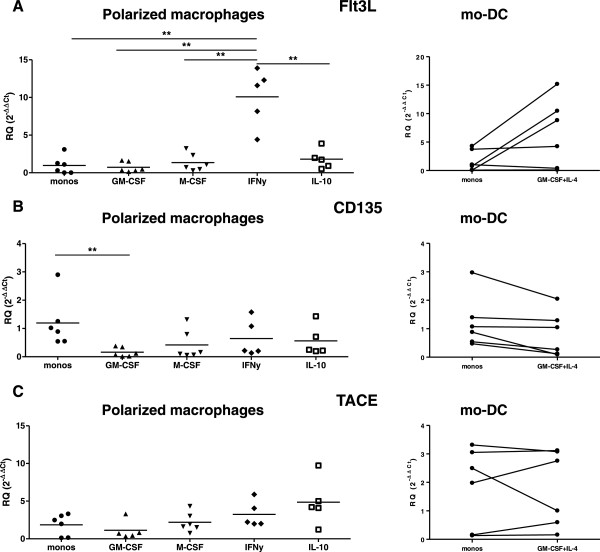
**Gene expression of FMS-related tyrosine kinase 3 ligand, CD135 and tumor necrosis factor-converting enzyme by *in vitro *polarized human macrophages and monocyte-derived dendritic cells.** Macrophages and monocyte-derived dendritic cells (mo-DCs) differentiated as stated in Methods. **(A)** Interferon-gamma (IFNγ)-differentiated macrophages expressed higher levels of FMS-related tyrosine kinase 3 ligand (Flt3L) compared with all the other polarizing conditions (IFNγ vs. monocytes, *P* = 0.0317; IFNγ vs. interleukin (IL)-10, *P* = 0.0079; IFNγ vs. macrophage colony-stimulating factor (M-CSF) and granulocyte–macrophage colony-stimulating factor (GM-CSF), *P* = 0.0043). There was a trend for higher Flt3L expression in mo-DC compared with monocytes but these differences did not reach statistical significance. **(B)** CD135 expression is higher in monocytes and is downregulated upon differentiation into macrophages. There was a trend for lower of CD135 expression in mo-DC compared with monocytes but these differences were not statistically significant. **(C)** Tumor necrosis factor-converting enzyme (TACE) expression was similar in monocytes and polarized macrophages and in monocytes and mo-DC. Bars represent mean ± standard error of the mean of at least five independent donors’ mRNA expression of each gene relative to Glyceraldehyde 3-phosphate dehydrogenase (GAPDH). *P <0.05, **P <0.01, ***P <0.001. monos.

The local environment plays a critical role in shaping or directing the pattern or pathway of macrophage and DC differentiation. To better understand the dynamics of Flt3L, CD135 and TACE expression with differentiation, we differentiated monocytes into macrophages using several polarizing conditions or into mo-DC and evaluated the expression of these markers. We observed that IFNγ-differentiated macrophages expressed higher levels of Flt3L compared with all the other polarizing conditions (Figure 5A; IFNγ vs. monocytes, *P* = 0.0317; IFNγ vs. IL-10, *P* = 0.0079; IFNγ vs. macrophage colony-stimulating factor and granulocyte–macrophage colony-stimulating factor, *P* = 0.0043). There was a trend towards higher Flt3L expression in mo-DC compared with monocytes but these differences did not reach statistical significance (Figure 5A). The expression level of CD135 tends to decrease with increasing differentiation [31]. Indeed, we observed that CD135 expression is highest in monocytes and downregulated upon differentiation into macrophages (Figure 5B). There was a trend towards lower CD135 expression levels in mo-DC compared with monocytes but these differences were not statistically significant (Figure 5B). TACE expression was similar in monocytes and polarized macrophages and in monocytes and mo-DC (Figure 5C).

### Flt3L serum levels in RA patients decrease after effective treatment

**Figure 6:**
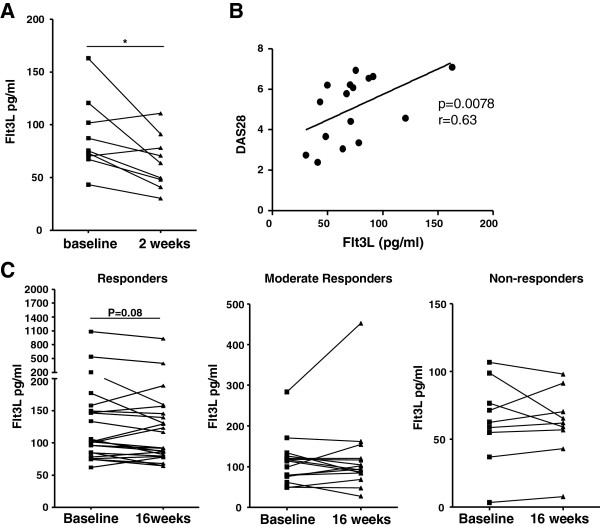
**FMS-related tyrosine kinase 3 ligand serum levels in response to prednisolone or adalimumab therapy in rheumatoid arthritis patients.** Serum was collected at baseline and post treatment, at 2 weeks in case of prednisolone treatment and 16 weeks for adalimumab treatment. Adalimumab patients were further discriminated between responders, moderate responders and nonresponders based on European League Against Rheumatism criteria. FMS-related tyrosine kinase 3 ligand (Flt3L) serum levels were assessed by enzyme-linked immunosorbent assay before and after therapy. **(A)** Serum levels of Flt3L decreased significantly (*P* = 0.02) after prednisolone treatment. **(B)** Positive correlation between the Disease Activity Score in 28 joints (DAS28) and Flt3L serum levels in prednisolone-treated patients (*P* = 0.0078, *r* = 0.63). **(C)** A trend towards lower serum levels of Flt3L was observed in RA patients (only responders) after adalimumab treatment. *P < 0.05.

Having shown increased levels of Flt3L in RA patients, we next asked the question of whether effective treatment could reduce Flt3L concentrations, which would suggest an effect on DCs associated with clinical improvement. Figure 6A shows significantly decreased Flt3L levels after prednisolone treatment (*P* = 0.02). There was a significant correlation between the Disease Activity Score in 28 joints and Flt3L serum levels (baseline and post treatment) (Figure 6B; *r* = 0.63, *P* = 0.0078). Similarly, there was a nonsignificant trend towards lower serum levels of Flt3L in RA patients responding to adalimumab treatment (Figure 6C).

## Discussion

In the present study we show in RA patients the expression of Flt3L and its receptor in three different compartments: blood, SF and ST. Flt3L was significantly elevated in RA SF compared with paired serum, confirming previous observations [23]. In addition, we reported for the first time that RA serum contains significantly elevated levels of Flt3L compared with HI serum. Moreover, we observed a higher expression of Flt3L (mRNA) in STs of RA patients compared with gout patients. RA and gout samples were matched in terms of macrophage numbers and IL-6 and IL-8 expression. This might indicate that Flt3L levels might reflect disease specificity more than just inflammation.

A detailed analysis of cellular components in paired blood and SF revealed that monocytes are the major cell population that expresses extracellular Flt3L. Monocytes are bone marrow-derived cells that mediate essential regulatory and effector functions in innate and adaptive immunity [32]. Circulating PB monocytes migrate into tissues, where they differentiate into different effector cells such as macrophages, DC and osteoclasts [33] that are of importance in RA pathology. The percentage of circulating CD14^+^ Flt3L^+^ monocytes is increased in RA patients, compared with HI. Moreover, in RA SF the percentage of Flt3L-expressing monocytes is significantly higher compared with paired PB. Importantly, RA SF monocytes have a superior capacity to express Flt3L on a per cell basis compared with PB (circulating) monocytes. Mobilization of preformed Flt3L from intracellular stores rather than *de novo* synthesis might be responsible for increased Flt3L levels at the site of inflammation. Flt3L is a crucial growth factor for DC differentiation that leads to increased numbers of these cells both in mice and humans [13]. Interestingly, Flt3L can substitute for macrophage colony-stimulating factor in support of osteoclast differentiation and function [34], raising the possibility of a direct role for Flt3L in bone damage in RA.

The expression of the receptor for Flt3L, CD135, is elevated in RA PB monocytes compared with HI PB monocytes. In addition, we show that RA SF monocytes express higher levels of CD135 compared with paired PB monocytes. Coexpression of CD135 and its ligand in the same cell (in this case by RA monocytes) suggests a possible autocrine stimulatory mechanism, as already reported for primary acute myeloid leukemia (AML) [35].

TACE is the major sheddase for Flt3L [15]. Flt3L is primarily produced as a membrane-bound protein [36] and soluble Flt3L is generated through ectodomain shedding [15]. TACE has been implicated in RA due to its role in processing membrane-bound TNF to its soluble form [37]. Previous studies have demonstrated a central role for TNF in RA, and early preclinical studies indicated that inhibition of TACE was beneficial for patients with arthritis [38]. We observed that in RA ST the main sources of TACE were macrophages (both CD68^+^ and CD163^+^ populations), CD55^+^ fibroblast-like synoviocytes, activated endothelial cells or infiltrating monocytes (CD31^+^) and B cells (CD19^+^). As soluble Flt3L levels are highly dependent on TACE activity, the above-mentioned cells might contribute to local levels of soluble Flt3L. Since the TACE expression level (mRNA) in RA and gout STs was the same and Flt3L levels were increased in RA ST, it is tempting to speculate that TACE biological activity in RA might be elevated compared with gout patients. This observation cannot be attributed to a differential degree of ST inflammation between RA and gout STs since the expression of the inflammatory cytokines IL-6 and IL-8 and macrophages numbers (CD68^+^ and CD163^+^ cells) was similar. Macrophages are critically involved in the pathogenesis of RA [39]. Not only do they produce a variety of proinflammatory cytokines and chemokines, but macrophages also contribute to cartilage and bone destruction [40]. In addition, it has been reported that the number of macrophages in RA ST correlates with bone damage and that increased numbers of macrophages are an early hallmark of active disease [3]. One of the main features of macrophages is their high plasticity during development. The nomenclature of general macrophage polarization has been proposed in the last decade, in which M1 (classical, inflammatory) and M2 (alternative, anti-inflammatory) refer to the two extremes of a spectrum of possible macrophage activation status [41]. M1 macrophages are mainly present in RA and are characterized by a proinflammatory phenotype, producing high levels of TNF, IL-1, IL-6, IL-12, reactive oxygen species, and low levels of IL-10 [42]. Here we show for the first time that, in addition to the above-mentioned inflammatory mediators, Flt3L might be considered a specific marker for IFNγ-differentiated macrophages. Polarizing cytokines such as IFNγ might contribute to the high levels of Flt3L found in RA synovium by shifting the macrophage polarization into a M1-like phenotype. Overall, these data suggest that in RA, in addition to circulating monocytes, IFNγ-differentiated macrophages might be an important source of Flt3L.

Sublining macrophages are a reliable biomarker for response to therapy in RA [43]. We have previously shown that oral prednisolone, an effective therapy in RA, was associated with a reduction in macrophage infiltration in ST [26]. In this study we observed a marked reduction of Flt3L serum levels in RA patients after prednisolone treatment, and a significant correlation between the Disease Activity Score in 28 joints and Flt3L serum levels. In addition, in the responder group of RA patients treated with adalimumab we observed a trend toward reduced serum levels of Flt3L. The reduction of Flt3L serum levels observed after effective treatment might reflect the reduction in the numbers of the main source(s) of Flt3L: circulating monocytes and/or ST macrophages. Flt3L has recently been outlined within a panel of preclinical biomarkers of predictive value for the development of RA [44]. Flt3L levels might therefore be valuable as a potential biomarker of inflammation or response to treatment.

## Conclusion

The Flt3L/CD135 axis is increased in RA patients compared with HI and disease controls, and is responsive to both prednisolone and adalimumab treatment. Collectively these data suggest that the Flt3L/CD135 axis might be important in RA pathophysiology.

## Abbreviations

DC: Dendritic cell; Flt3L: FMS-related tyrosine kinase 3 Ligand; HI: Healthy individuals; IFN: Interferon; IL: Interleukin; mDC: Myeloid dendritic cell; MFI: Mean fluorescence intensity; mo-DC: Monocyte-derived dendritic cell; NK: Natural killer; PB: Peripheral blood; PBMC: Peripheral blood mononuclear cells; pDC: Plasmacytoid dendritic cell; qPCR: Quantitative polymerase chain reaction; RA: Rheumatoid arthritis; SF: Synovial fluid; ST: Synovial tissue; TACE: Tumor necrosis factor-converting enzyme; TNF: Tumor necrosis factor.

## Competing interests

The authors declare that they have no competing interests.

## Authors’ contributions

MIR, PPT and MCL were responsible for study conception and design. MIR, SGP, SA, BH and PB were responsible for acquisition of data. MIR, SGP, DMG, KAR and MCL were responsible for analysis and interpretation of data. MIR and MCL drafted the manuscript. All authors revised the manuscript critically for important intellectual content and approved the final version.

## Supplementary Material

Additional file 1 Contains a description of the methods from Additional files 2, 3 and 4. Click here for file

Additional file 2: Figure S1 Assessment of global ST inflammation. (A-B) IL-6 and IL-8 gene expression level in STs from RA (n=22) and gout (n=12) patients. Gene expression analysis by qPCR showed that IL-6 and IL-8 expression was similar between RA ST compared to gout ST. Each data point represents a single subject. Results are presented as mean±SEM mRNA expression of IL-6 or IL-8 relative to GAPDH. *p < 0.05, **p < 0.01. (C-D) Immunohistochemical analysis of CD68 and CD163 macrophage markers in RA (n=12), gout (n=11) and HI (n=7) STs. CD68 and CD163 macrophage numbers were increased in RA and gout STs compared to HI STs. No differences were observed between RA and gout STs. Click here for file

Additional file 3: Figure S2 Characterization of Flt3L expression in RA paired PBMC and SFMC and in HI PBMC. Intra- and extra-cellular expression of Flt3L by all cell types is shown in terms of percentage or MFI (cellular marker+ Flt3L+). No differences were observed for the percentage or MFI of extracellular or intracellular Flt3L by CD4+ T cells (A), CD8+ T cells (B) or CD19+ B cells (C) in RA compared to HI. Bars represent the mean (±SEM) of 5-10 RA patients and HI. *p < 0.05, **p < 0.01. Click here for file

Additional file 4: Figure S3 Immunofluorescence staining of TACE^+^ cells in RA ST. (A) Single stainings for TACE^+^ (red) and other cellular markers (green) can be seen. (B) Double immunofluorescence staining of TACE^+^ cells in RA ST with CD3^+^ T cells and vWF^+^ blood vessels. TACE did not colocalized with CD3^+^ T cells or with vWF^+^ blood vessels. Single immunofluorescence stainings for TACE and vWF are also shown. Figures are representative of five RA patients. Original magnification 250x. Click here for file
