# Supplementary material for: FMS-related tyrosine kinase 3 ligand (Flt3L)/CD135 axis in rheumatoid arthritis
Source: Arthritis Res Ther. 2013 Dec 6;15(6):R209. doi: 10.1186/ar4403 (PMC3978611; doi:10.1186/ar4403)
Supplement: Additional file 1 — Contains a description of the methods from Additional files 2, 3 and 4. [file ar4403-S1.doc]

**Supplementary Methods**

**Immunophenotyping by FACS**

Single-cell suspensions were stained with the indicated fluorochrome-conjugated antibodies for surface and intracellular stainings. The folowing antibodies were used: purified goat anti human Flt3L (Santa Cruz, Heidelberg, Germany), allophycocyanin (APC)- conjugated anti-CD304 (Miltenyi Biotec, Bergisch Gladbach, Germany), Alexa 700-conjugated anti-CD19 (Becton Dickinson (BD), Breda, The Nederlands), phycoerythrin (PE)–conjugated anti-CD135 (eBioscience, Vienna, Austria), eFluor Alexa 750-conjugated anti-CD14 (BD), biotin-conjugated anti-CD1C (Miltenyi), PercP Cy5,5-conjugated anti-CD123 (eBioscience), PE-Cy7–conjugated anti-CD56 (BD), PerCP-Cy5.5–conjugated anti-CD4 (BD), APC-Cy7–conjugated anti-CD3 (BD), Alexa 610–conjugated anti-CD8 ((Invitrogen, Breda, The Netherlands) and APC-conjugated anti-CD94 (BD). Intracellular stainings were performed for Flt3L and CD135 after surface staining with the above indicated fluorochome-conjugated antibodies after fixation (4% paraformaldehyde) and permeabilization (saponin, Sigma Aldrich, Zwijndrecht, The Netherlands). Cytometry data were acquired with a FACS CANTO (Becton Dickinson) and analyzed with Flow Jo Flow Cytometry Analysis software (Tree Star, Ashland, OR). Results were expressed as the percentage of positive cells and mean fluorescence intensity (MFI).

**Quantitative measurement of mRNA expression**

Total RNA was isolated from synovial tissue biopsy samples using RNA Stat-60 (Tel-Test, Friendswood, TX) then treated with DNase I (Invitrogen), and reverse transcribed using RevertAid H Minus First-Strand cDNA synthesis kit (Fermentas, St. Leon-Rot, Germany). RNA from GM-CSF, M-CSF, IFN-γ, IL-10-differentiated macrophages and RNA from mo-DC was isolated using the RNeasy Kit and RNase-Free DNase Set (Qiagen, Venlo, The Netherlands). 500 ng of total RNA was reverse-transcribed using SuperScript™ II RT (Invitrogen). The RNA concentration was determined with a NanoDrop spectrophotometer. Duplicate PCR reactions were performed using SYBR green (Applied Biosystems, Foster City, CA) with an ABI Prism® 7000 sequence detection system (Applied Biosystems). cDNA was amplified using specific primers: Flt3L forward, GGAGCCCAACAACCTATCTC; Flt3L Reverse, CTGTGTTGGAAGGAGCAGTC; CD135 we used primer mix from Qiagen PPH00804F-200; TACE forward, ACTGGACCACCAGAGAATGG; TACE reverse, GGCCAAACCACACAAGAACT, IL-6 forward, GACAGCCACTCACCTCTTCA; IL-6 reverse, CCTCTTTGCTGCTTTCACAC; IL-8 forward, GCTCTGTGTGAAGGTGCAGT; IL-8 reverse, CCAGACAGAGCTCTCTTCCA and GAPDH forward, TTCACCACCATGGAGAAG, GAPHD reverse, GGCATGGACTGTGGTCAT.

All PCR data were normalized to the expression of GAPDH, used as an internal control. PCR data were obtained as Ct values and the mean of the duplicate Ct values of each sample was calculated. Relative levels of gene expression were normalised to GAPDH housekeeping gene (HK) using the comparative Ct method.
