# Supplementary figures and images for: FMS-related tyrosine kinase 3 ligand (Flt3L)/CD135 axis in rheumatoid arthritis
Source: Arthritis Res Ther. 2013 Dec 6;15(6):R209. doi: 10.1186/ar4403 (PMC3978611; doi:10.1186/ar4403)

## Slide 1
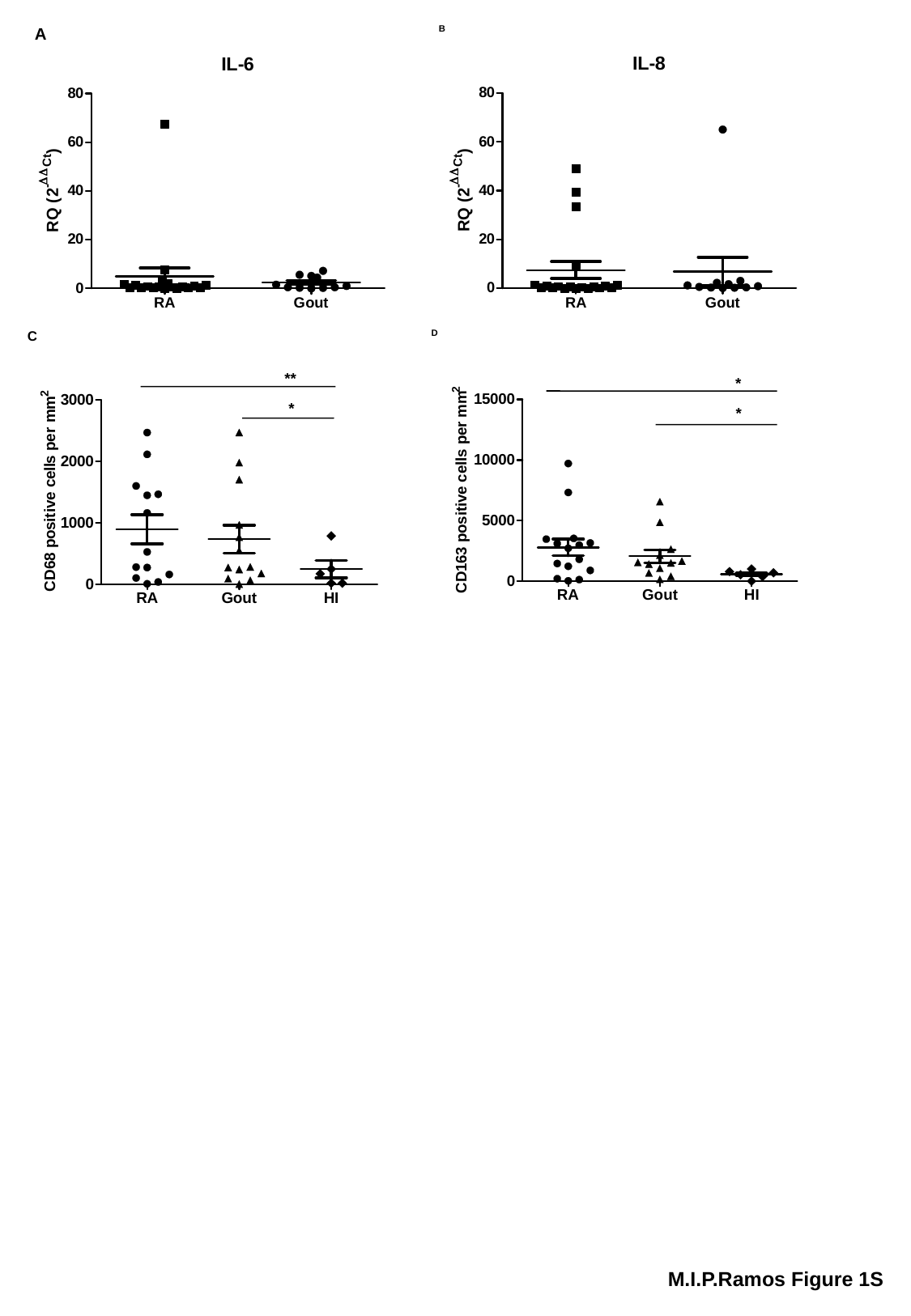

A
B
C
D
**
*
*
*
M.I.P.Ramos Figure 1S

Supplement: Additional file 2: Figure S1 — Assessment of global ST inflammation. (A-B) IL-6 and IL-8 gene expression level in STs from RA (n=22) and gout (n=12) patients. Gene expression analysis by qPCR showed that IL-6 and IL-8 expression was similar between RA ST compared to gout ST. Each data point represents a single subject. Results are presented as mean±SEM mRNA expression of IL-6 or IL-8 relative to GAPDH. *p < 0.05, **p < 0.01. (C-D) Immunohistochemical analysis of CD68 and CD163 macrophage markers in RA (n=12), gout (n=11) and HI (n=7) STs. CD68 and CD163 macrophage numbers were increased in RA and gout STs compared to HI STs. No differences were observed between RA and gout STs. [file ar4403-S2.ppt]

## Slide 1
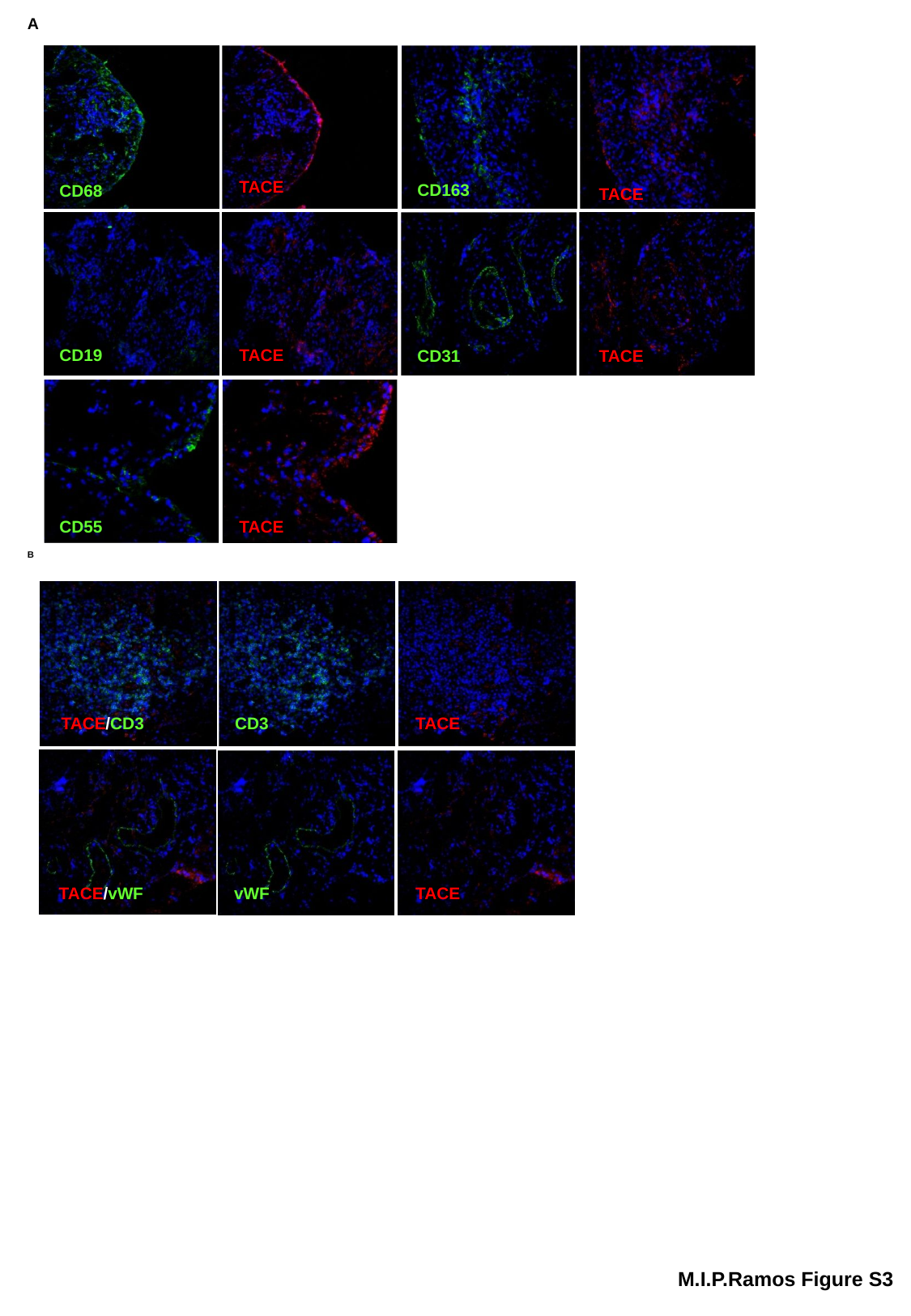

A
CD68
TACE
CD163
TACE
CD19
TACE
CD31
TACE
CD55
TACE
B
TACE/CD3
CD3
TACE
TACE/vWF
vWF
TACE
M.I.P.Ramos Figure S3

Supplement: Additional file 4: Figure S3 — Immunofluorescence staining of TACE+ cells in RA ST. (A) Single stainings for TACE+ (red) and other cellular markers (green) can be seen. (B) Double immunofluorescence staining of TACE+ cells in RA ST with CD3+ T cells and vWF+ blood vessels. TACE did not colocalized with CD3+ T cells or with vWF+ blood vessels. Single immunofluorescence stainings for TACE and vWF are also shown. Figures are representative of five RA patients. Original magnification 250x. [file ar4403-S4.ppt]
