# Supplementary material for: FMS-related tyrosine kinase 3 ligand (Flt3L)/CD135 axis in rheumatoid arthritis
Source: Arthritis Res Ther. 2013 Dec 6;15(6):R209. doi: 10.1186/ar4403 (PMC3978611; doi:10.1186/ar4403)
Supplement: Additional file 3: Figure S2 — Characterization of Flt3L expression in RA paired PBMC and SFMC and in HI PBMC. Intra- and extra-cellular expression of Flt3L by all cell types is shown in terms of percentage or MFI (cellular marker+ Flt3L+). No differences were observed for the percentage or MFI of extracellular or intracellular Flt3L by CD4+ T cells (A), CD8+ T cells (B) or CD19+ B cells (C) in RA compared to HI. Bars represent the mean (±SEM) of 5-10 RA patients and HI. *p < 0.05, **p < 0.01. [file ar4403-S3.ppt]

## Slide 1
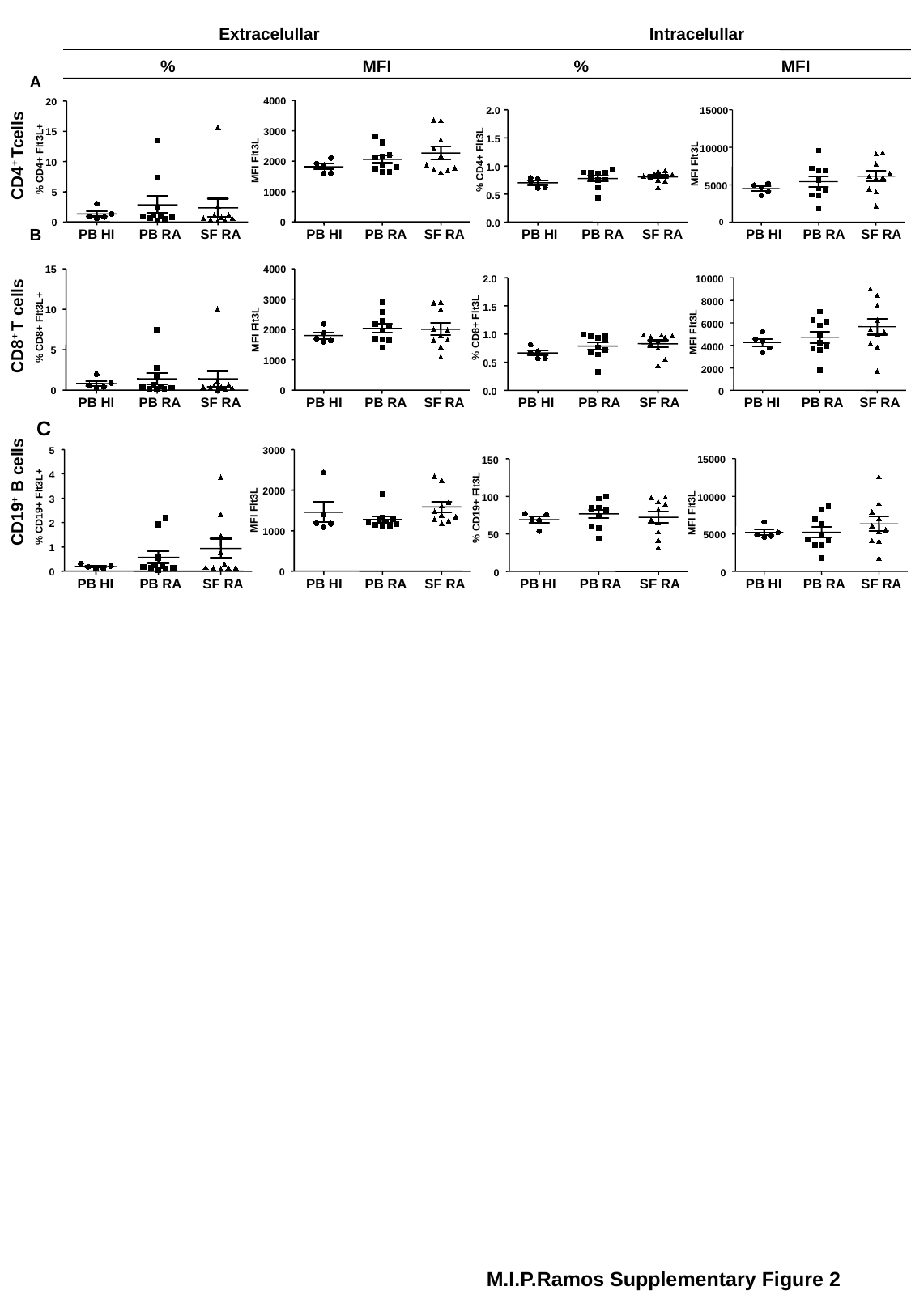

Extracelullar
Intracelullar
%
MFI
%
MFI
A
4000
3000
MFI Flt3L
2000
1000
0
PB HI
PB RA
SF RA
20
15
% CD4+ Flt3L+
10
5
0
PB HI
PB RA
SF RA
2.0
1.5
% CD4+ Flt3L
1.0
0.5
0.0
PB HI
PB RA
SF RA
15000
10000
MFI Flt3L
5000
0
PB HI
PB RA
SF RA
CD4+ Tcells
B
15
10
% CD8+ Flt3L+
5
0
PB HI
PB RA
SF RA
4000
3000
MFI Flt3L
2000
1000
0
PB HI
PB RA
SF RA
2.0
1.5
% CD8+ Flt3L
1.0
0.5
0.0
PB HI
PB RA
SF RA
10000
8000
6000
MFI Flt3L
4000
2000
0
PB HI
PB RA
SF RA
CD8+ T cells
C
5
4
3
% CD19+ Flt3L+
2
1
0
PB HI
PB RA
SF RA
3000
2000
MFI Flt3L
1000
0
PB HI
PB RA
SF RA
15000
10000
MFI Flt3L
5000
0
PB HI
PB RA
SF RA
150
100
% CD19+ Flt3L
50
0
PB HI
PB RA
SF RA
CD19+ B cells
M.I.P.Ramos Supplementary Figure 2
